# Supplementary material for: Quantifying Variation in Bacterial Reproductive Fitness: a High-Throughput Method
Source: mSystems. 2021 Feb 2;6(1):e01323-20. doi: 10.1128/mSystems.01323-20 (PMC7857537; doi:10.1128/mSystems.01323-20)
Supplement: TEXT S1 [file mSystems.01323-20-s0001.docx]

# Supplement A – BaColonyzer Description

### Grid Location Detection

BaColonyzer starts the analysis by finding the location of the grid. For that, it imports the grayscale version of the last image in the time-lapse series, which is used to generate a histogram of colours. Colours are represented as intensity values ranging from 0 to 255.

Since the last image contains well-defined colony spots, the histogram usually shows two high peaks: the highest peak corresponds to the colour of the agar while the second peak represents the colour of the colonies. As a quality check, BaColonyzer ensures that the colour intensity of the colonies falls between 70% of the maximum colour of the picture (upper bound) and twice the colour of the agar (lower bound). Otherwise, it sets the colour intensity of the colonies to be within this range.

The intensity values of agar and spots are used to create an artificial image of the plate (template), for which users need to input the number of rows and columns of the grid. Resizing and changing the position of this template allows to find the best match within the actual grayscale image. This is achieved by computing the lowest normalised squared difference.

Once the best match is found, BaColonyzer is able to predict the location of the whole plate and to trim those parts of the image that might introduce noise (e.g., the borders). Image trimming, followed by automatic thresholding using Otsu’s binarization, allows to get the position of the spots and the agar. In order to ensure that pixels belonging to the colonies are never considered as agar, dilation of colony spots is also performed. For that, the dilation kernel is considered to be 5% of the colony area.

### Image Analysis

Each of the images in series is imported in grayscale and trimmed based on the grid location that has been previously derived.

By default, colour intensities of the image are also normalised (divided by 255) to be in range 0-1. Alternatively, in order to compare different series of time-lapse images, BaColonyzer allows the users to provide a reference picture for the normalization. This must be an image showing a white and black area next to each other, and must be taken using the same camera settings as the image series. The reference image is imported in grayscale and the colour intensities are clipped at 1%-99% quantiles to exclude outliers or noise. The resulting minimum and maximum intensity values (black and white, respectively) are taken to calibrate the colour intensities of images using the expression below:

$$NI= \frac{{OI-min}_{ref}}{{max}_{ref}-{min}_{ref}}$$

where NI are the normalised intensity values of each image, OI are the original intensity values, min_ref_ is the intensity of the black colour, and max_ref_ is the intensity of the white colour.

Next, to ensure an accurate analysis of each colony, BaColonyzer divides the agar plate into smaller patches that only contain one colony spot. By default, the tool adjusts the intensity values of each patch by subtracting the mean intensity of the agar in this patch, thus correcting for colour differences within and between images.

At the end, BaColonyzer provides a result table with statistics of each colony, including normalised intensities (NI) of each patch, colony area, colony mean, colony variance, background mean, and background variance. Results are stored as Output Data. Furthermore, in order to visually check that the grid location was achieved properly, BaColonyzer provides some output Images. These are binary images resulting from an Otsu’s Binarization, which is performed after the trimming step to let the users check whether the grid localisation was successful.

### BaColonyzer Output

Upon using BaColonyzer with the time lapse photographs of a given agar plate, two folders are automatically created in the photograph directory, one named “Output Data”, and a second named “Output Images”.

The folder “Output Data” contains tab delimited text files showing the image metrics computed by BaColonyzer. Each file provides information of one image. Files are named exactly the same as the original image file, ending with ".out". The output parameters for each image and each colony area in these files are listed in Supplement A Table I.

The “Output Images” folder contains binary images (black/white), which are named exactly the same as the original image files used for the analysis. These binary images can be used to visually check the performance of BaColonyzer to detect the position of the plate, the agar and the colonies on the solid growth media. Thus, black pixels show the agar and white pixels correspond with detected bacterial growth. In addition, the borders of the original pictures are deleted in order to take only the surface of the agar plate (growth area) and ignore the rest.
