# Supplementary figures and images for: Quantifying Variation in Bacterial Reproductive Fitness: a High-Throughput Method
Source: mSystems. 2021 Feb 2;6(1):e01323-20. doi: 10.1128/mSystems.01323-20 (PMC7857537; doi:10.1128/mSystems.01323-20)

**A**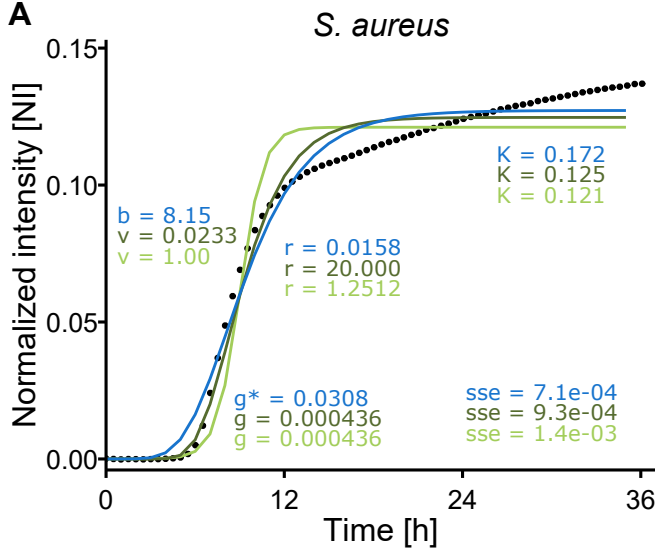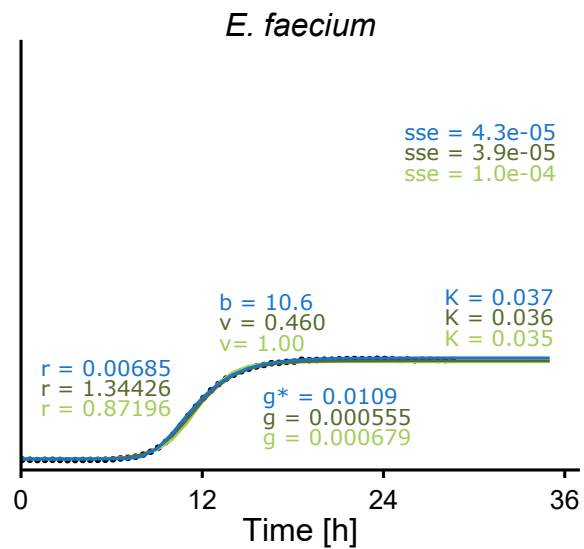**B**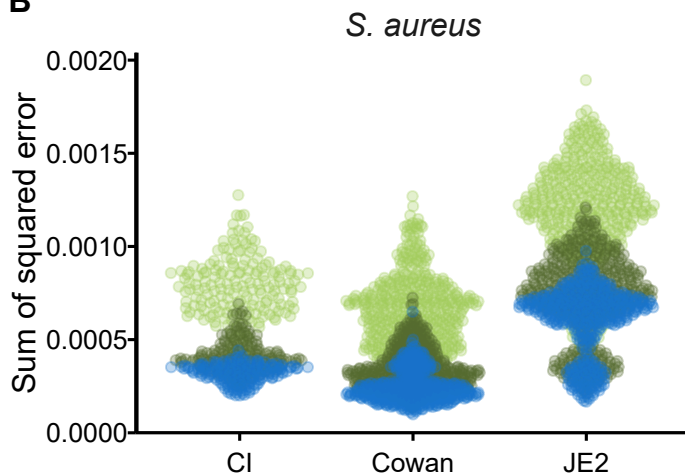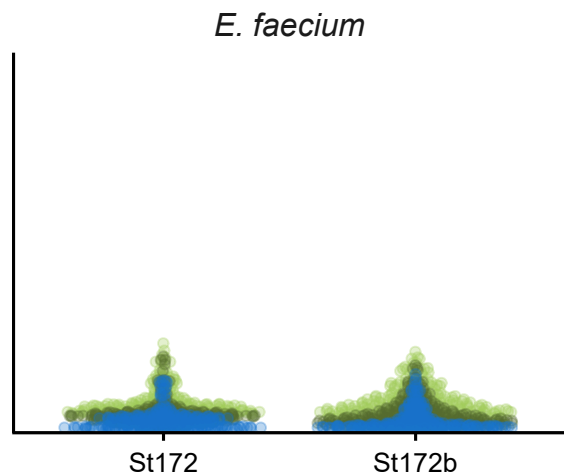

Supplement: FIG S1 [file mSystems.01323-20-sf001.pdf]

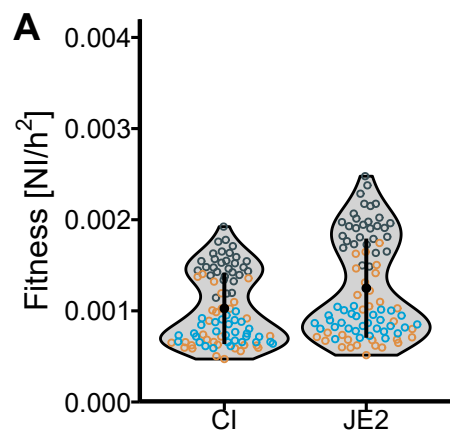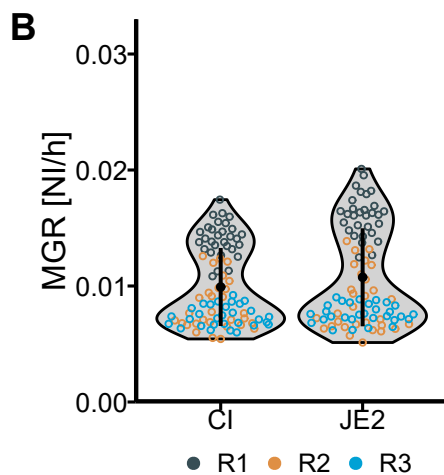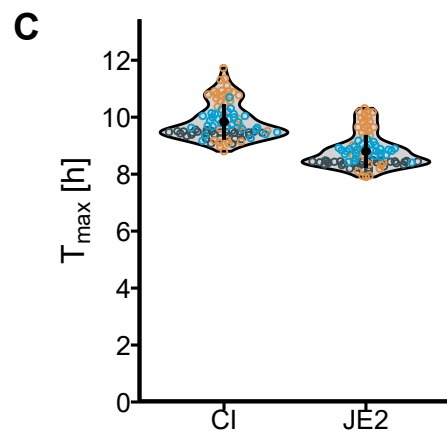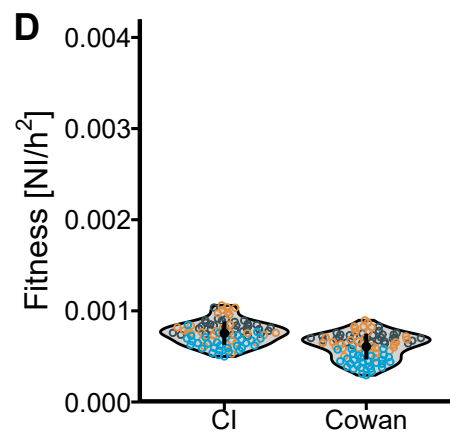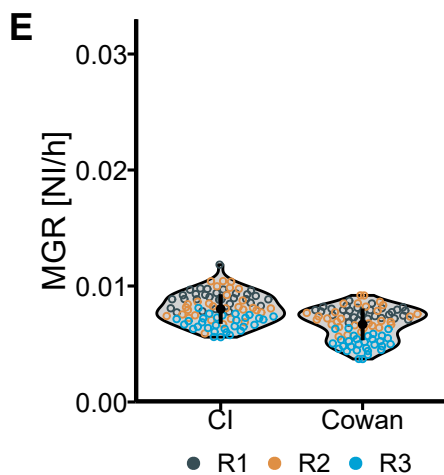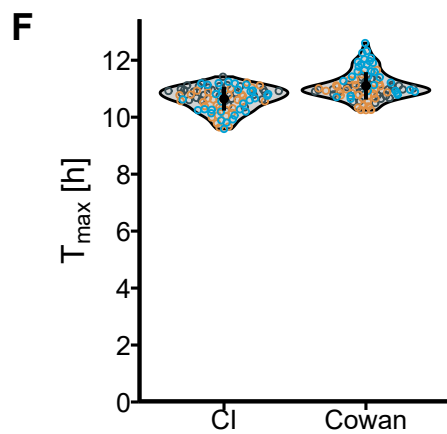

Supplement: FIG S2 [file mSystems.01323-20-sf002.pdf]

*S. aureus*

Grid

Solo

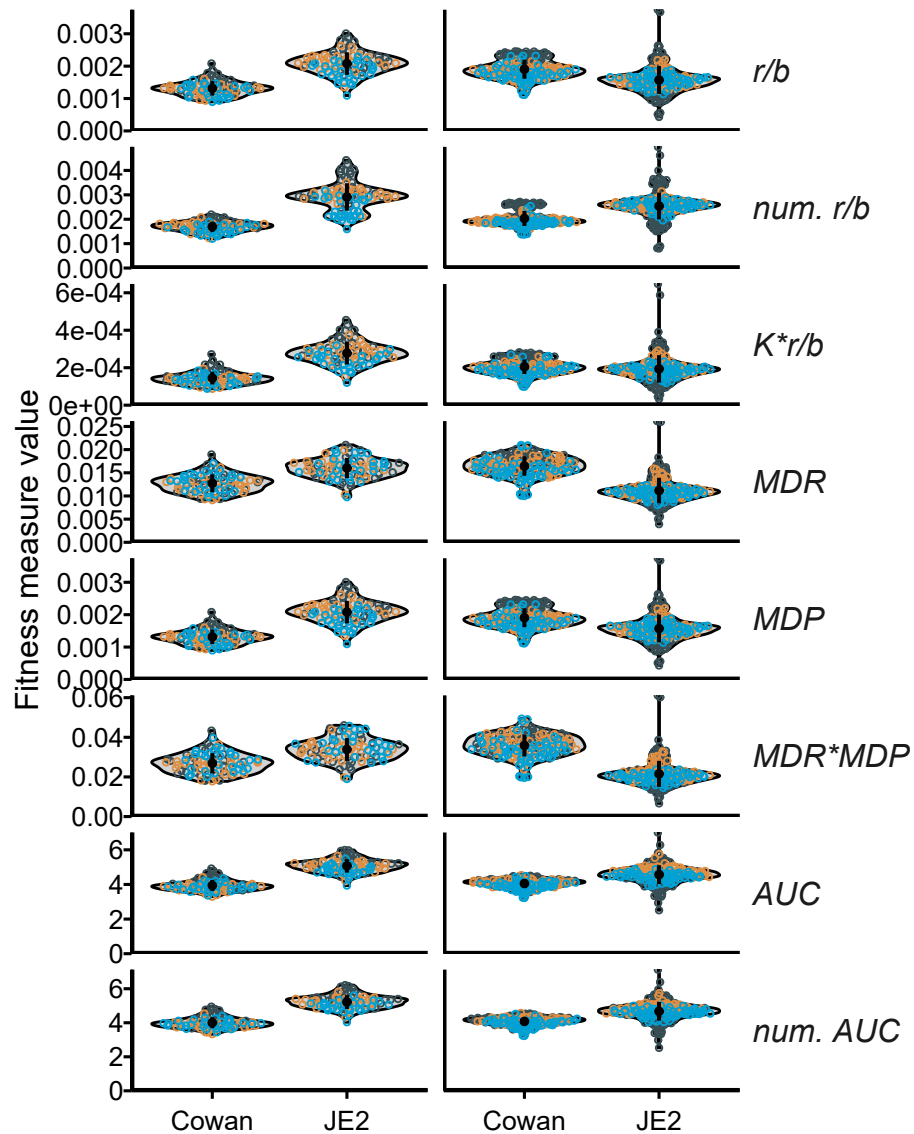*E. faecium*

Grid

Solo

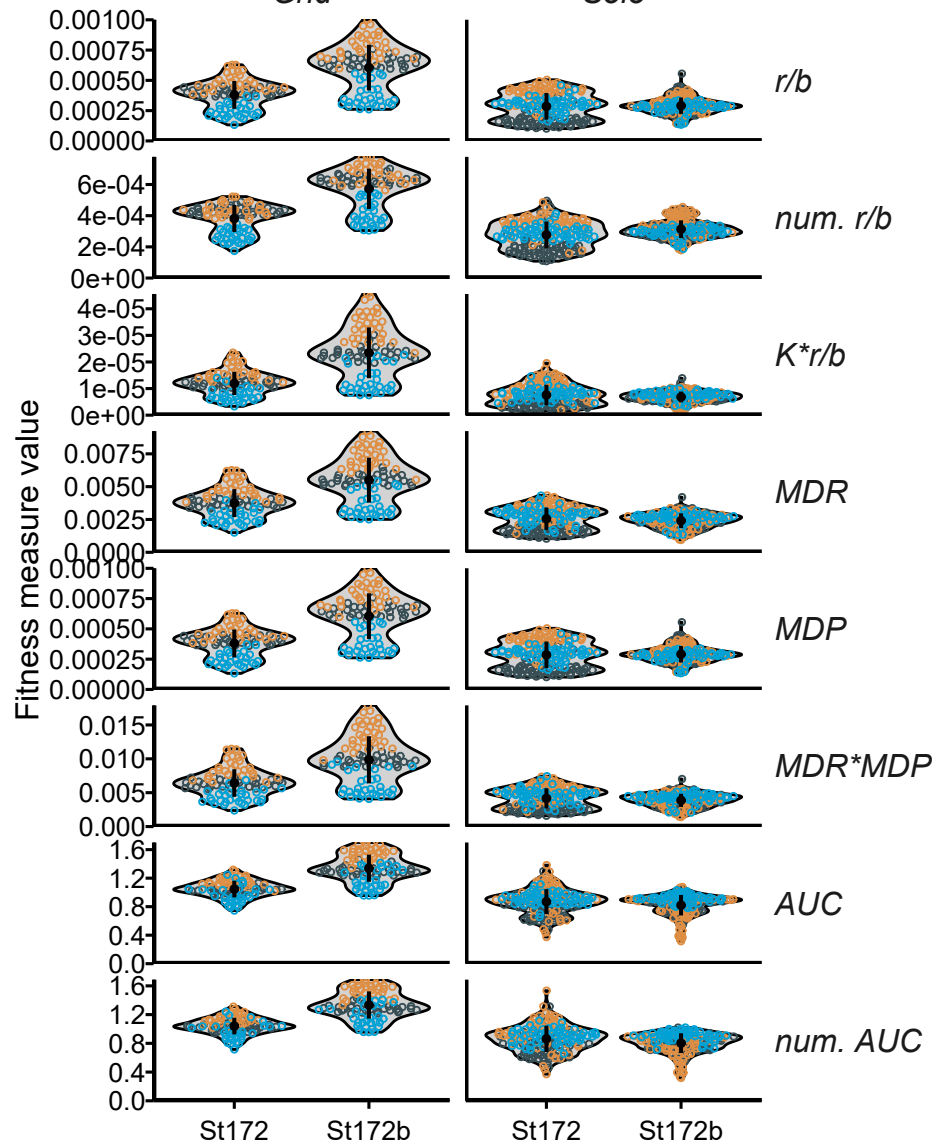

Supplement: FIG S3 [file mSystems.01323-20-sf003.pdf]

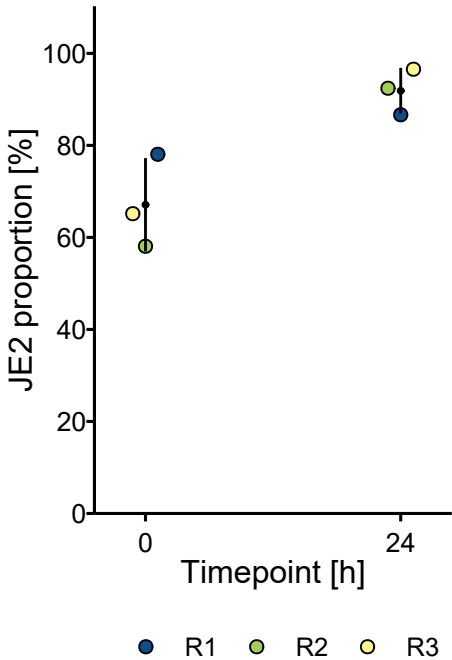

Supplement: FIG S4 [file mSystems.01323-20-sf004.pdf]

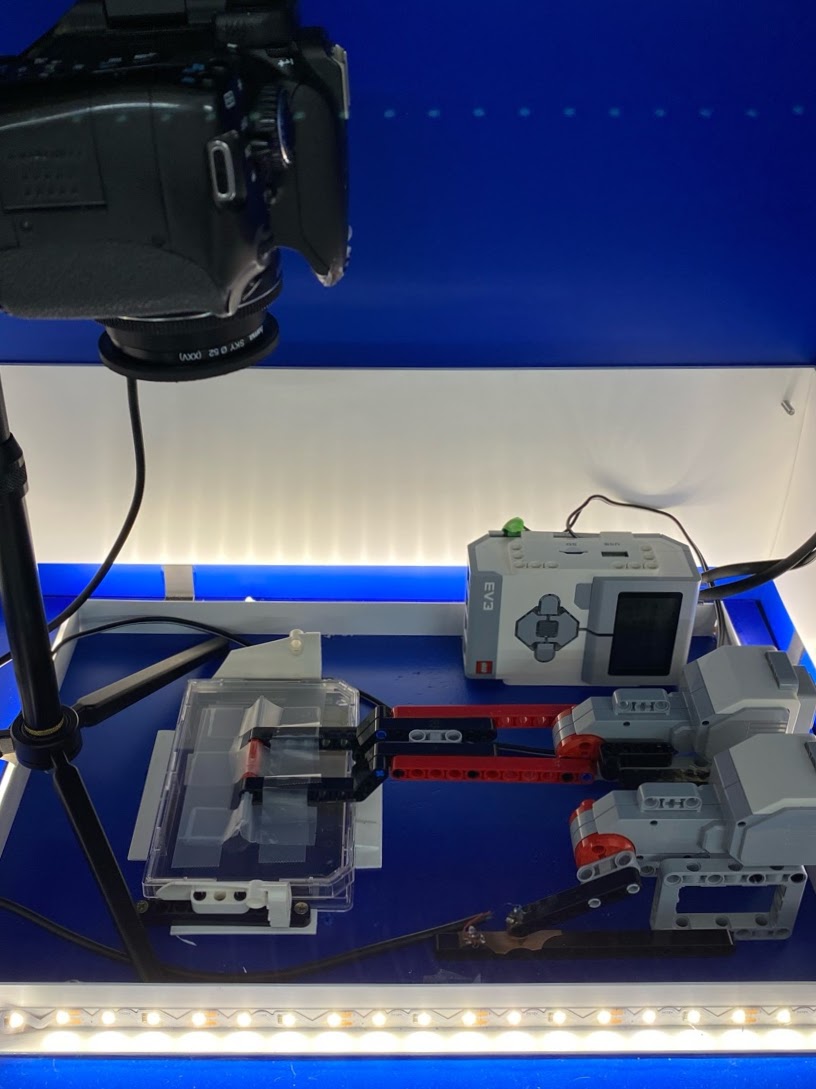

Supplement: FIG S5 [file mSystems.01323-20-sf005.jpg]

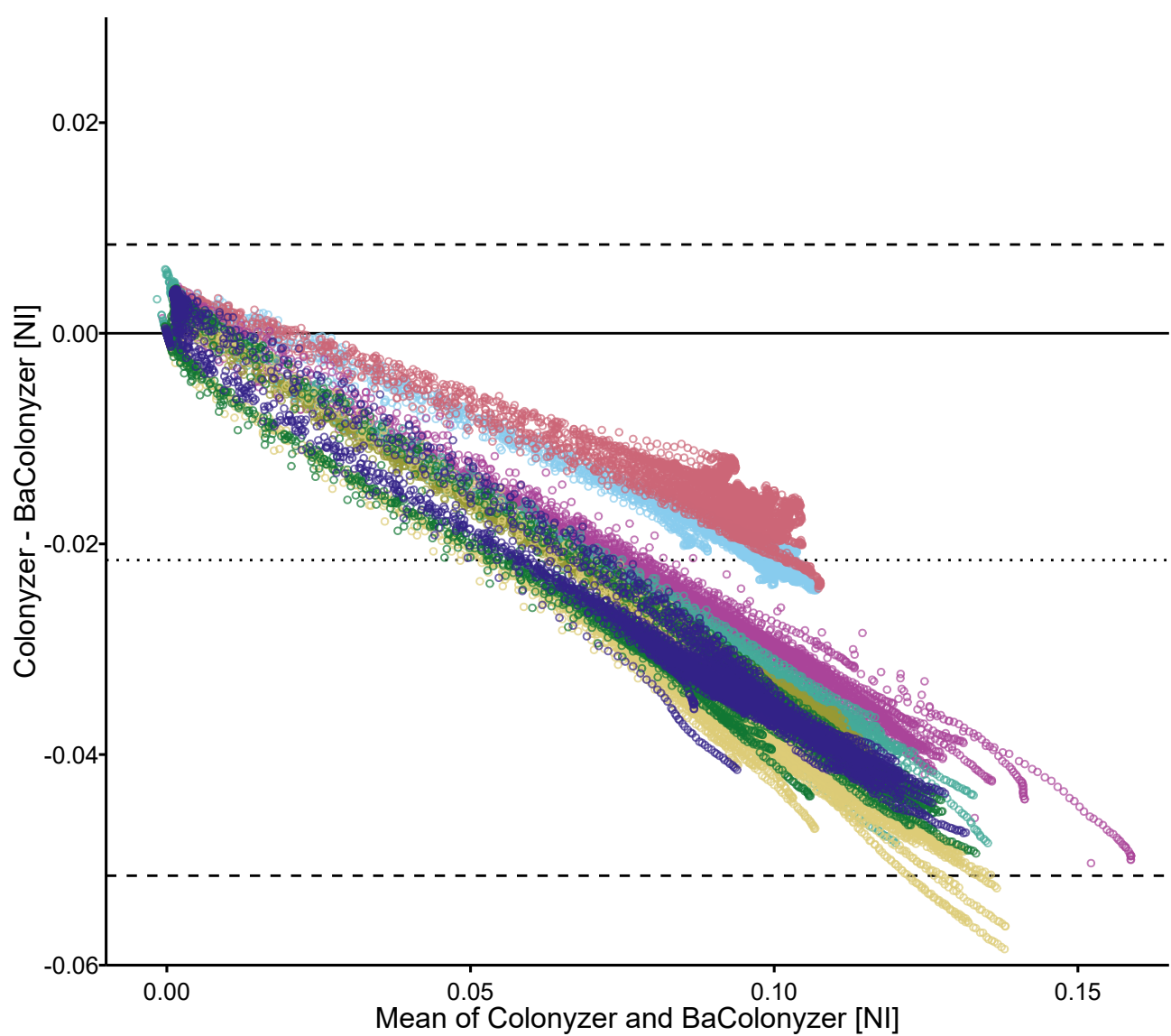

Supplement: FIG S6 [file mSystems.01323-20-sf006.pdf]
