# Supplementary material for: Quantifying Variation in Bacterial Reproductive Fitness: a High-Throughput Method
Source: mSystems. 2021 Feb 2;6(1):e01323-20. doi: 10.1128/mSystems.01323-20 (PMC7857537; doi:10.1128/mSystems.01323-20)
Supplement: TABLE S1 [file mSystems.01323-20-st001.docx]

### Supplement A Table I: BaColonyzer Output Parameters

| **Parameter** | **Description** |
| --- | --- |
| Row | Row of each colony in the agar plate. |
| Column | Column of each colony in the agar plate. |
| Intensity | Total normalised light intensity (NI) of the colony. This value is used as the cell density estimate for future calculations. |
| Area | Area of the colony. |
| Colony Mean | Mean intensity values of the colony. |
| Colony Variance | Variance of the intensity values of the colony. |
| Background mean | Mean intensity values of the agar (without growth) in each patch. |
| Barcode | Prefix that is used to name all images in the series. |
| File name | Complete name of this image in the series. |
